# Preoperative neutrophil-to-lymphocyte ratio predicts the surgical outcome of Xp11.2 translocation/TFE3 renal cell carcinoma patients

**DOI:** 10.1186/s12894-018-0374-z

**Published:** 2018-06-11

**Authors:** Sezim Agizamhan, Feng Qu, Ning Liu, Jing Sun, Wei Xu, Lihua Zhang, Hongqian Guo, Weidong Gan

**Affiliations:** 1 Department of Urology, Nanjing Drum Tower Hospital, The Affiliated Hospital of Nanjing University Medical School, No. 321 Zhongshan Road, Nanjing, 210008 Jiangsu Province China; 2 Department of Oncology, Jiangsu Province Hospital, The First Affiliated Hospital of Nanjing Medical University, Nanjing, Jiangsu China; 3 Department of Pathology, Jiangsu Cancer Hospital, The Affiliated Cancer Hospital of Nanjing Medical University, Nanjing, Jiangsu China; 4 Department of Pathology, Zhongda Hospital Southeast University, Nanjing, Jiangsu China

**Keywords:** Neutrophil-to-lymphocyte ratio, C-reactive protein/albumin ratio, Platelet-to-lymphocyte ratio, Renal cell carcinoma, Xp11.2 translocation

## Abstract

**Background:**

The preoperative neutrophil-to-lymphocyte ratio (NLR), C-reactive protein/albumin ratio (CRP/Alb ratio) and platelet-to-lymphocyte ratio (PLR) have been demonstrated to predict the clinical outcome of various human cancer, including renal cell carcinoma(RCC). The aim of our study was to explore the prognostic values of these ratios in patients with Xp11.2 translocation/TFE3 gene fusions renal cell carcinoma (Xp11.2 tRCC).

**Methods:**

A retrospective multicentre study was performed in 82 Xp11.2 tRCC patients who underwent radical or partial nephrectomy. The optimal cutoff values of the NLR, CRP/Alb ratio and PLR were determined by the receiver operating characteristic (ROC) analysis. The impact of the NLR, CRP/Alb ratio and PLR, as well as other clinicopathological characteristics, on disease-free survival (DFS) and overall survival (OS) were evaluated using the univariate and multivariate Cox regression analyses.

**Results:**

The optimal cutoff values of the NLR, CRP/Alb ratio and PLR were set at 2.45, 140 and 0.08, respectively, according to the ROC analysis. Univariate analyses showed that the NLR, CRP/Alb ratio and PLR all were associated with DFS of Xp11.2 tRCC patients (*P* < 0.001, *P* = 0.005 and *P* = 0.001, respectively) and OS of Xp11.2 tRCC patients (*P* = 0.016, *P* = 0.003 and *P* = 0.014, respectively). Multivariate analysis indicated that the NLR was independently associated with DFS of Xp11.2 tRCC patients (hazard ratio [HR]: 4.25; 95% confidence interval [95% CI]: 1.19–15.18; *P* = 0.026) along with age (*P* = 0.004), the pT status (*P* < 0.001) and the pN status (*P* < 0.019), and the NLR (HR: 26.26; 95% CI: 1.44–480.3; *P* = 0.028) also was independently associated with OS in patients with Xp11.2 tRCC, along with age (*P* = 0.016) and a tumour thrombus (*P* = 0.007).

**Conclusion:**

Overall, relatively high NLRs, CRP/Alb ratios and PLRs were associated with a poor prognosis of Xp11.2 tRCC patients; among of them, only the NLR independently predicted the progression of Xp11.2 tRCC, and the NLR may help to identify patients with high metastasis or relapse risk.

## Background

Xp11.2 translocation/TFE3 gene fusions renal cell carcinoma (Xp11.2 tRCC) was first listed as a new type of renal cell carcinoma (RCC) in the 2004 by the World Health Organization (WHO). Since then, it has received wide attention [1–5]. Xp11.2 tRCC is characterized by various translocations of the transcription factor E3(TFE3) on chromosome Xp11.2, resulting in overexpression of the TFE3 protein [6]. Xp11.2 tRCC is a kind of relatively rare tumour that predominantly occurs in children and young adults [7]. Regardless of its low incidence, Xp11.2 RCC is more aggressive than conventional RCC because most adult present at advanced stages and invasive clinical courses [3, 4]. Therefore, it is crucial to identify new preoperative prognostic factors to provide additional prognostic information for Xp11.2 tRCC patients. In addition, in regard to the risk of disease recurrence, is important to obtain prognostic information in the preoperative phase for the postoperative surveillance and possible adjuvant therapy.

It is recognized that the inflammatory processes in the tumour microenvironment play a significant role in promoting the proliferation, invasion and metastasis of the malignant cells [8, 9]. Inflammatory markers, such as the neutrophil count(NC), lymphocyte count(LC), platelet count (PLT), neutrophil-to-lymphocyte ratio (NLR), C-reactive protein (CRP), albumin (Alb), C-reactive protein/ albumin ratio (CRP/Alb ratio) and platelet-to-lymphocyte ratio (PLR), have been shown to predict the clinical outcome of various human cancers [10–13]. For renal cell carcinoma, several publications demonstrated that high NLR, CRP/Alb ratios and PLRs were associated with a poor prognosis in RCC, respectively [14–17].

To our knowledge, the prognostic value of inflammatory markers has never been investigated in the Xp11.2 tRCC patients. Additionally, compared with conventional RCC, Xp11.2 tRCC involves different genetic characteristics and biological pathways and is associated with a more worse prognosis [3, 5, 18]. In addition, inflammatory markers are more easily accessible than other prognostic factors before surgery. Therefore, there is a need to identify new preoperative prognostic markers to predict the clinical outcomes of surgical Xp11.2 tRCC patients. The aims of the present study were to examine the prognostic values of the NLR, CRP/Alb ratio and PLR in patients with Xp11.2 tRCC.

## Methods

### Patients

Institutional review board approval was obtained at Nanjing Drum Tower Hospital, Jiangsu Province Hospital, Jiangsu Cancer Hospital and Zhongda Hospital Southeast University for this multicentre retrospective study. All the patients have provided informed written consents to have their medical record data used in research. Between January 2007 and July 2017, 89 consecutive patients from the 4 institutions described above who were diagnosed with Xp11.2 tRCC after radical or partial nephrectomy for a renal mass were reviewed for the present study. All clinicopathological data were retrieved from medical records from the department of urology as well as from pathology reports from the Institute of Pathology at each institution. The inclusion criteria included the following: 1) patients who were histologically and immunohistochemically (using the TFE3 protein nuclear stain) diagnosed with Xp11.2 tRCC; 2) the data on complete blood laboratory tests included the serum neutrophil count(NC), lymphocyte count(LC), platelet count (PLT), C-reactive protein (CRP) level and albumin (Alb) level within one week before performing radical or partial nephrectomy; and (3) patients without blood laboratory tests before surgery, patients with active inflammatory disease and patients with other tumours were excluded from the study. Finally, a total of 82 patients were enrolled in this study.

### Clinical and pathological evaluation

**Fig. 1:**
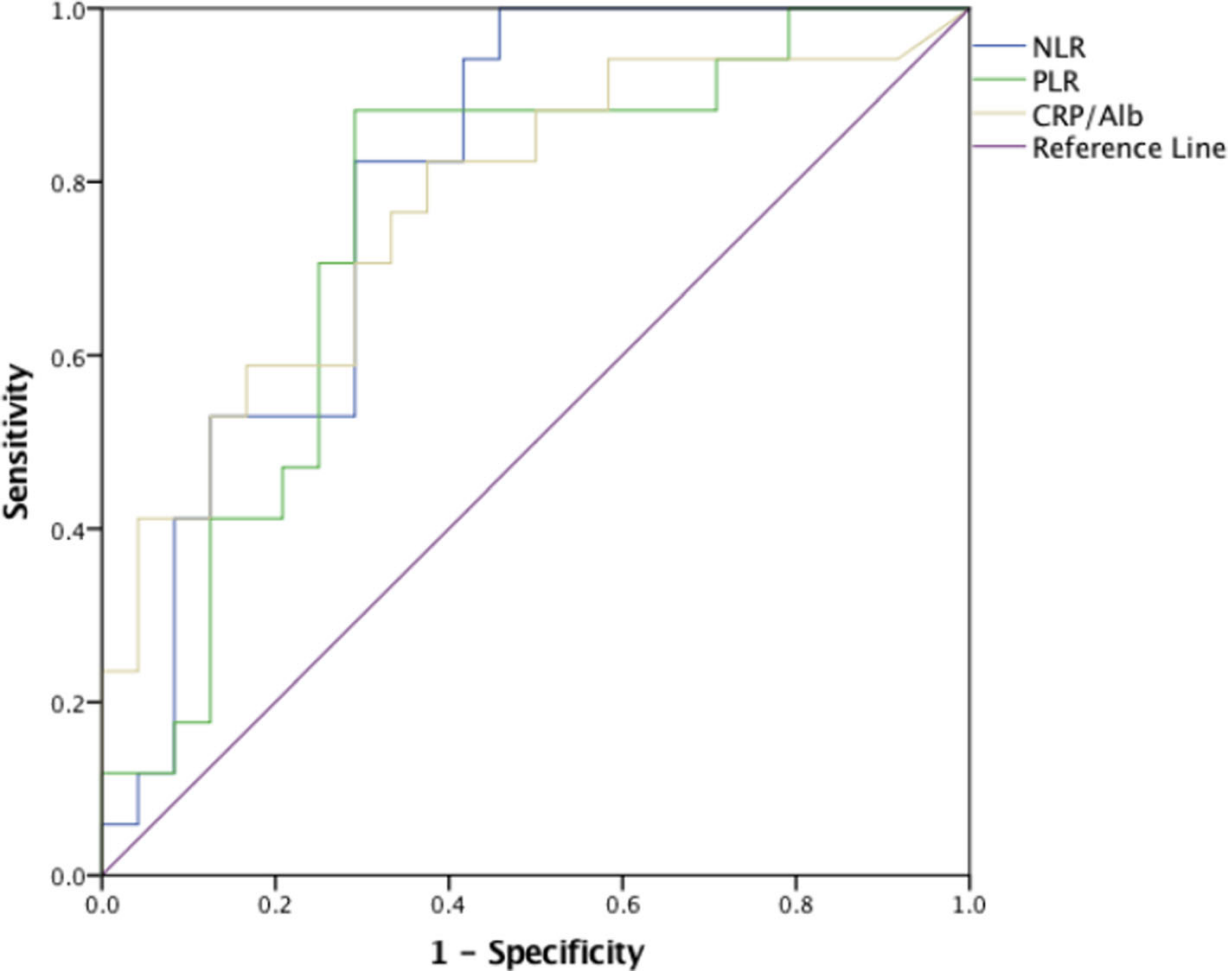
The predictive abilities of the preoperative NLR, CRP/Alb ratio and PLR were compared using ROC curves

The baseline clinical characteristics and pathologic information, including data on the age at the time of surgery, gender, tumour location, tumour size, symptoms at presentation, surgical treatment, pathological features, immunohistochemistry results, NC, LC, PLT, CRP level, Alb level, lactate dehydrogenase (LDH) level, urine protein, tumour stage, and nuclear grade, were all collected. Tumour stage was determined according to the seventh edition of the TNM-UICC/AJCC classification system, and the nuclear grade was defined based on the Fuhrman Grading System. The NLR was defined as the ratio of the NC to LC. The PLR was defined as the ratio of the PLT to LC. The CRP/Alb ratio was defined as the ratio of the serum CRP level to the serum albumin (Alb) level. Elevated LDH was defined as serum LDH > 245 U/L. The association between inflammatory parameters (LN, NC, PLT, CRP, NLR, PLR, CRP/Alb) and DFS was explored, and the ROC curves of the NLR, CRP/Alb ratio and PLR are shown in Fig. 1.

### Patients follow-up

All patients enrolled in this study were followed-up every 3 months during the first 2 years, every 6 months for 3–5 years and every 12 months after 5 years until July 2017 or until death. A physical examination, laboratory tests, and dynamic computed tomography were performed at every visit. Overall survival (OS) was defined as the time interval between the date of surgery and the date of death or the last follow-up. Disease-free survival (DFS) was defined as the time interval between the date of surgery and date of disease recurrence or metastasis or the last follow-up in localized Xp11.2 tRCC patients who underwent radical or partial nephrectomy.

### Statistical analyses

Statistical analyses were performed using SPSS version 24.0 software (SPSS, Chicago, IL, USA). The descriptive data (i.e., tumour size) were presented as the means ± standard deviation or medians, and Student’s t-test was used for these variables. A comparison between groups was performed using the Chi-squared test. Receiver operating characteristic (ROC) analysis was used for the selection of effective inflammatory parameters and corresponding optimal cut-off values, and the area under the curve (AUC), sensitivity, specificity and *P* value were calculated accordingly. Survival analyses of OS and DFS were performed using the Kaplan-Meier method, and the log-rank test was performed for the significance comparison. A Cox proportional-hazard model was applied for univariate and multivariate analyses, and hazard ratios (HR) with 95% confidence intervals (95% CI) and *P* values are presented. If variables were significantly associated with other variables, they were excluded from the final multivariable analysis. A *P* value < 0.05 was considered statistically significant in all statistical analyses.

## Results

### Patient and tumour characteristics

The clinicopathological characteristics of 82 Xp11.2 tRCC patients are shown in Table 1. Among them, 78 (95.1%) were diagnosed with a localized mass (T1–3 N0/+ M0), while 4 (4.9%) had distant metastasis before surgery. After a median follow-up time of 31 months (range: 2 to 108 months), 14 (17%) died and 34 (41%) developed recurrence or distant metastasis. Their mean age at surgery was 37 years (range: 2 to 71 months). The 5-year overall survival (OS) was 82.9% (68/82), 5-year-cancer-specific survival (CSS) was 86.6% (71/82), and 5-year disease-free survival (DFS) was 61.5% (48/78).

**Table 1:** Baseline characteristics of 82 Xp11.2 tRCC patients

Characteristic	Case (*n* = 82)
Age in years	
> 45	21
≤ 45	61
Sex	
Male	33
Female	49
Tumour size, cm	5.94 ± 2.65
Location	
Left	31
Right	51
Symptoms	
Asymptomatic	48
Symptomatic	34
Fuhrman grade	
1 to 2	32
3 to 4	50
TNM stage	
I to II	49
III to IV	33
pT status	
T1 to T2	64
T3 to T4	18
pN status	
N0	59
N1	23
pM status	
M0	78
M1	4
Surgical treatment	
Radical	55
Partial	27
Tumour thrombus	
Negative	72
Positive	10

### Optimal cut-off values of inflammatory parameters based on ROC analysis

Based on ROC analysis, the area under ROC curve (AUC) values of the NLR, CRP/Alb ratio, and PLR were 0.797 (*P* = 0.001), 0.772 (*P* = 0.003), and 0.755 (*P* = 0.006), respectively, for DFS and the optimal cut-off values for the NLR, CRP/Alb, and PLR were 2.45, 0.83, and 140, respectively. The corresponding sensitivity and specificity values for DFS were, respectively, 82.4 and 72.8% for NLR, 70.6 and 72.8% for CRP/Alb, and 80.0 and 65.9% for PLR.

### Association of the preoperative NLR and clinicopathological characteristics

Stratified by the cut-off value, the association between the preoperative NLR and clinicopathological characteristics is summarized in Table 2. An elevated NLR was significantly associated with the tumour size (*P* < 0.001), Fuhrman-grade (*P* = 0.011), TNM stage (*P* < 0.001), pT status (*P* = 0.001), pN status (*P* < 0.001), tumour thrombus (*P* = 0.014), CRP/Alb (*P* < 0.001), PLR (*P* = 0.001), LDH (*P* = 0.014) and proteinuria (*P* < 0.013). For patients in the high NLR group, only 35.0% of patients were at stage I/II and 65.0% of patients were at stage III-IV (*P* < 0.001), 62.5% of patients were at stage T1/T2, and 37.5% of patients were at stage T3/T4 (*P* = 0.001). However, for patients in the low NLR group, 83.3% of patients were at stage I/II, 16.7% were at stage III-IV (*P* < 0.001), 92.9% of patients were at stage T1/T2, and 7.1% of patients were at stage T3/T4 (*P* = 0.001). Meanwhile, the percentage values of patients at stage N0/N1 were 52.5% / 47.2%, and the percentage values of patients negative/positive tumour thrombus were 77.5%/22.5% in the high NLR group. By comparison, the percentage values of patients at stage N0/N1 were 90.5%/9.5%, and the percentage values of patients with negative/positive tumour thrombus were 97.6%/2.4% in the low NLR group. These results revealed that a high NLR was associated with tumour progression and a low NLR was associated with early-stage of Xp11.2 tRCC. Similarly, Clinicopathological features of Xp11.2 RCC patients stratified by the cut-off value of the CRP/Alb ratio, PLR are summarized in the Table 3.

**Table 2:** Clinicopathological features of Xp11.2 tRCC patients stratified by the cut-off value of the NLR

Characteristic	N (%)	NLR > 2.45 (*n* = 40)	NLR ≤ 2.45 (*n* = 42)	*P*
Age in years				0.004
> 45	21 (25.6%)	16 (40.0%)	5 (11.9%)	
≤ 45	61 (74.4%)	24 (60.0%)	37 (88.1%)	
Sex				0.345
Male	33 (40.2%)	14 (35.0%)	19 (45.2%)	
Female	49 (59.8%)	26 (65.0%)	23 (54.8%)	
Tumour size	5.94 ± 2.65	7.0 ± 3.0	4.0 ± 1.1	< 0.001
Location				0.377
Left	31 (37.8%)	18 (45.0%)	23 (54.8%)	
Right	51 (62.2%)	22 (55.0%)	19 (45.2%)	
Symptoms				0.477
Asymptomatic	48 (58.5%)	25 (62.5%)	23 (54.8%)	
Symptomatic	34 (41.5)	15 (37.5%)	19 (45.2%)	
Fuhrman grade				0.011
1 to 2	32 (39.0%)	10 (25%)	22 (52.4%)	
3 to 4	50 (61.0%)	30 (75%)	20 (47.6%)	
TNM stage				< 0.001
I-II	49 (59.8%)	14 (35.0%)	35 (83.3%)	
III-IV	33 (40.2%)	26 (65.0%)	7 (16.7%)	
pT status				0.001
T1-T2	64 (78.0%)	25 (62.5%)	39 (92.9%)	
T3-T4	18 (22.0%)	15 (37.5%)	3 (7.1%)	
pN status				< 0.001
N0	59 (72.0%)	21 (52.5%)	38 (90.5%)	
N1	23 (28.0%)	19 (47.5%)	4 (9.5%)	
pM status				0.112
M0	78 (95.1%)	36 (90.0%)	42 (100.0%)	
M1	4 (4.9%)	4 (10.0%)	0 (0.0%)	
Surgical treatment				0.307
Radical	55 (67.1%)	29 (72.5%)	26 (61.9%)	
Partial	27 (32.9)	11 (27.5)	16 (38.1%)	
Tumour thrombus				0.014
Negative	72 (87.8%)	31 (77.5%)	41 (97.6%)	
Positive	10 (12.2%)	9 (22.5%)	1 (2.4%)	
CRP/Alb				< 0.001
≤ 0.083	44 (53.7%)	13 (32.5%)	31 (73.8%)	
> 0.083	38 (46.3%)	27 (67.5%)	11 (26.2%)	
PLR				0.001
≤ 140	46 (56.1%)	15 (37.5%)	31 (73.8%)	
> 140	36 (43.9%)	25 (62.5%)	11 (26.2%)	
LDH				0.014
Normal	68 (82.9%)	29 (72.5%)	39 (92.9%)	
Elevated	14 (17.1%)	11 (27.5%)	3 (7.1%)	
Proteinuria				0.013
No	63 (76.8%)	26 (65.0%)	37 (88.1%)	
Yes	19 (23.2%)	14 (35.0%)	5 (11.9%)

**Table 3:** Clinical-pathological features of Xp11.2 tRCC patients stratified by the cut-off values of the CRP/Alb ratio and PLR

Characteristic	N (%)	CRP/Alb> 0.083(*n* = 39)	CRP/Alb≤0.083(*n* = 43)	*P*	PLR > 140 (*n* = 36)	PLR ≤ 140 (*n* = 46)	*P*
Age in years				0.011			0.015
> 45	21 (25.6%)	15 (38.5%)	6 (14.0%)		14 (38.9%)	7 (15.2%)	
≤ 45	61 (74.4%)	24 (61.5%)	37 (86.0%)		22 (61.1%)	39 (84.8%)	
Sex				0.224			0.113
Male	33(40.2%)	13(33.3%)	20(46.5%)		11 (30.6%)	22 (47.8%)	
Female	49(59.8%)	26(66.7%)	23(53.5%)		25 (69.4%)	24 (52.2%)	
Tumor size	5.94 ± 2.65	6.7 ± 3.0	4.8 ± 2.1	0.035	7.4 ± 3.1	4.1 ± 1.2	< 0.001
Location				0.052			0.858
Left	31(37.8%)	19 (48.7%)	12 (27.9%)		14 (38.9%)	17 (37.0%)	
Right	51(62.2%)	20 (51.3%)	31 (72.1%)		22 (61.1%)	29 (63.0%)	
Symptoms				0.412			0.165
Asymptomatic	48 (58.5%)	21 (53.8%)	27 (62.8%)		18 (50.0%)	30 (65.2%)	
Symptomatic	34 (41.5%)	18 (46.2%)	16 (37.2%)		18 (50.0%)	16 (34.8%)	
Fuhrman grade				0.018			0.006
1 to 2	32 (39.0%)	10 (25.6%)	22 (51.2%)		8 (22.2%)	24 (52.2%)	
3 to 4	50 (61.0%)	29 (74.4%)	21 (48.8%)		28 (77.8%)	22 (47.8%)	
TNM stage				0.004			< 0.001
I-II	49 (59.8%)	17 (43.6%)	32 (74.4%)		12 (33.3%)	37 (80.4%)	
III-IV	33 (40.2%)	22 (56.4%)	11 (25.6%)		24 (66.7%)	9 (19.6%)	
pT status				0.018			0.006
T1-T2	64 (78.0%)	26 (66.7%)	38 (88.4%)		22 (61.1%)	42 (91.3%)	
T3-T4	18 (22.0%)	13 (33.3%)	5 (11.6%)		14 (38.9%)	4 (8.7%)	
pN status				0.003			0.003
N0	59 (72.0%)	22 (56.4%)	37 (86.0%)		20 (55.6%)	39 (84.8%)	
N1	23 (28.0%)	17 (43.6%)	6 (14.0%)		16 (44.4%)	7 (15.2%)	
pM status				0.101			0.199
M0	78 (95.1%)	35 (89.7%)	43 (100.0%)		33 (91.7%)	45 (97.8%)	
M1	4 (4.9%)	4 (10.3%)	0 (0.0%)		3 (8.3%)	1 (2.2%)	
Surgical treatment				0.502			0.686
Radical	55 (67.1%)	23 (59.0%)	22 (51.2%)		25 (69.4%)	30 (65.2%)	
Partial	27 (32.9)	16 (41.0%)	11 (48.8%)		11 (30.6%)	16 (34.8%)	
Tumour thrombus				0.011			0.001
Negative	72 (87.8%)	30 (76.9%)	42 (97.7%)		26 (72.2%)	46 (100.0%)	
Positive	10 (12.2%)	9 (23.1%)	1 (2.3%)		10 (27.8%)	0 (0.0%)	
CRP/Alb				–			< 0.001
≤ 0.083	44 (53.7%)	–	–		11 (30.6%)	33 (71.7%)	
> 0.083	38 (46.3%)	–	–		25 (69.4%)	13 (28.3%)	
PLR				0.009			–
≤ 140	46(56.1%)	16 (41.0%)	30 (69.8%)		–	–	
> 140	36(43.9%)	23 (59.0%)	13 (30.2%)		–	–	
LDH				0.011			0.023
Normal	68(82.9%)	28 (71.8%)	40 (93.0%)		26 (72.2%)	42 (91.3%)	
Elevated	14(17.1%)	11 (28.2%)	3 (7.0%)		10 (27.8%)	4 (8.7%)	
Proteinuria				< 0.001			< 0.001
No	63(76.8%)	22 (56.4%)	41 (95.3%)		20 (55.6%)	43 (93.5%)	
Yes	19 (23.2%)	17 (43.6%)	2 (4.7%)		16 (44.4%)	3 (6.5%)	

### Univariate and multivariate analyses for both DFS and OS

The results of univariate and multivariate analyses for both DFS and OS are shown in Tables 4 and 5. Univariate analysis demonstrated that age, the Fuhrman grade, pT status, pN status, tumour thrombus, the NLR, the CRP/Alb and the PLR were significant predictors for both DFS and OS in Xp11.2 tRCC patients. Multivariable analysis showed that the NLR (HR: 4.25, 95%, CI 1.19–15.18, *P* = 0.026) was an independent predictor of DFS in patients with Xp11.2 tRCC, along with pT status (*P* < 0.001), pT status (*P* = 0.019) and age (*P* = 0.004), and the NLR (HR: 26.26; 95% CI: 1.44–480.3; *P* = 0.028) also was an independent predictor of OS in patients with Xp11.2 tRCC, along with age (*P* = 0.016) and a tumour thrombus (*P* = 0.007).

**Table 4:** Univariate and multivariate analyses for variables considered for disease-free survival (DFS) (Cox proportional hazard regression model) (*n* = 78)

Variables	Univariate analysis		Multivariate analysis	
	HR (95% CI)	*P*	HR (95% CI)	*P*
Age (> 45)	2.34 (1.03 to 5.30)	*0.043*	6.25 (1.78 to 21.97)	*0.004*
Symptoms (yes)	1.06 (0.51 to 2.19)	0.885		
Gender (male)	0.76 (0.36 to 1.64)	0.490		
Fuhrman Grade (G3–G4)	5.24 (2.12 to 12.96)	*< 0.001*	1.83 (0.64 to 5.24)	0.261
pT status (T3-T4)	6.48 (2.96 to 14.19)	*< 0.001*	6.84 (2.35 to 19.90)	*< 0.001*
pN status (N1)	5.21 (2.52 to 10.77)	*< 0.001*	3.40 (1.22 to 9.43)	*0.019*
Tumour thrombus (yes)	12.47 (4.81 to 32.34)	*< 0.001*	2.90 (0.73 to 11.48)	0.129
NLR (> 2.45)	4.98 (2.12 to 11.66)	*< 0.001*	4.25 (1.19 to 15.18)	*0.026*
CRP/Alb (> 0.083)	2.90 (1.37 to 6.13)	*0.005*	1.40 (0.43 to 4.54)	0.574
PLR (> 140)	3.76 (1.74 to 8.13)	*0.001*	1.36 (0.47 to 3.72)	0.598

Italicized *P* values are statistically significant

**Table 5:** Univariate and multivariate analyses for variables considered for overall survival (OS) (Cox proportional hazard regression model) (*n* = 82)

Variables	Univariate analysis		Multivariate analysis	
	HR (95% CI)	*P*	HR (95% CI)	*P*
Age (> 45 years)	4.90(1.44 to 16.69)	*0.011*	26.56 (1.85 to 380.7)	*0.016*
Symptoms (yes)	2.04 (0.70 to 5.93)	0.189		
Gender (male)	2.14 (0.48 to 9.63)	0.320		
Fuhrman Grade (G3–G4)	1.92 (1.04 to 3.55)	*0.037*	0.30 (0.07 to 1.28)	0.103
pT status (T3-T4)	2.21 (1.24 to 3.95)	*0.007*	1.82 (0.70 to 4.70)	0.217
pN status (N1)	6.22 (2.06 to 18.82)	*0.001*	2.03 (0.20 to 20.45)	0.547
Tumour thrombus (yes)	22.32 (6.76 to 73.72)	*< 0.001*	47.40 (2.92 to 769.9)	*0.007*
NLR (> 2.45)	4.46 (1.33 to 14.97)	*0.016*	26.26 (1.44 to 480.3)	*0.028*
CRP/Alb (> 0.083)	23.51(2.90 to 190.51)	*0.003*	6.65 (0.35 to 127.0)	0.208
PLR (> 140)	4.30 (1.34 to 13.82)	*0.014*	0.14 (0.01 to 1.67)	0.120

Italicized *P* values are statistically significant

### The relationships of the preoperative NLR, pT status, pN status, age and tumour thrombus with survival

**Fig. 2:**
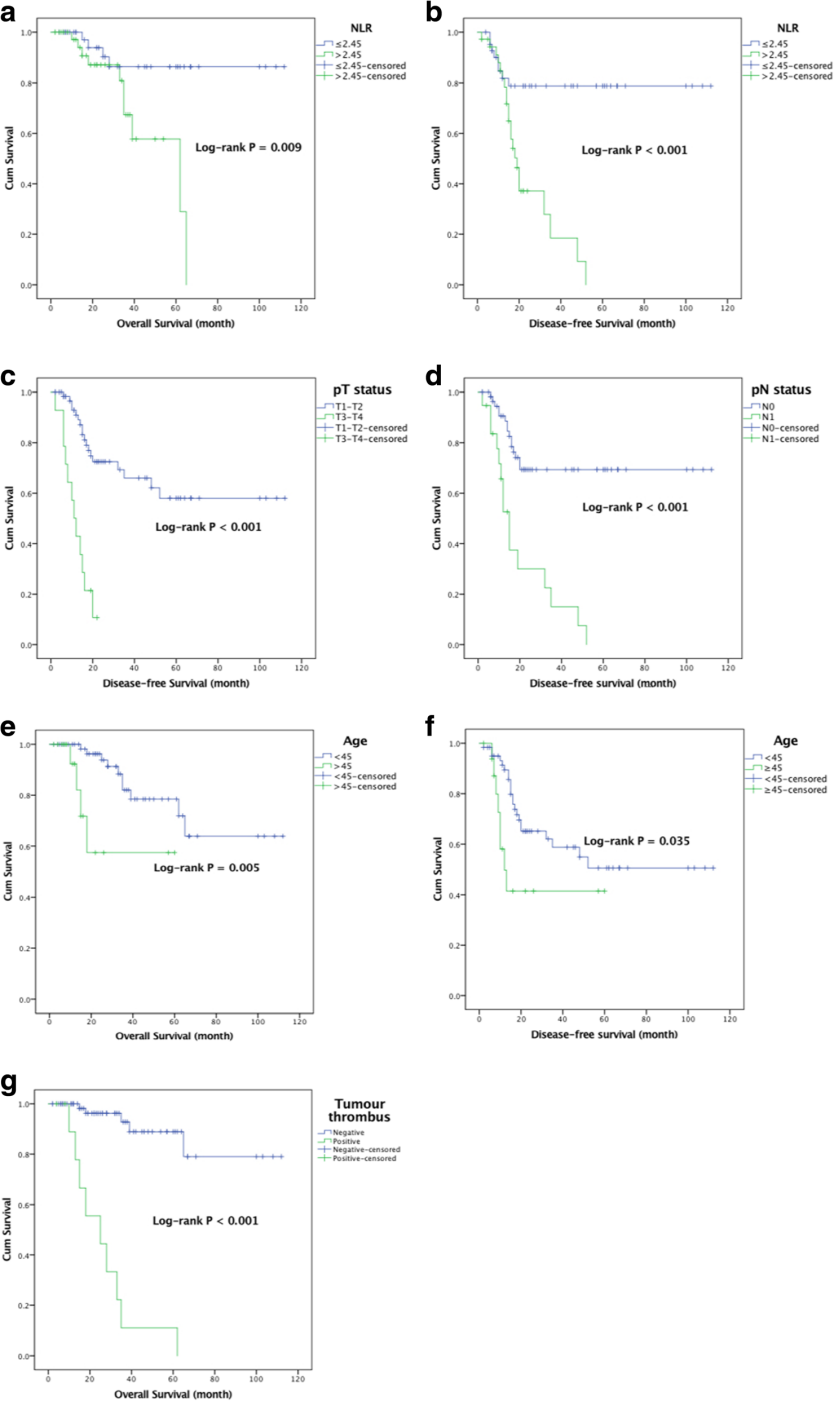
Kaplan–Meier curves for independent predictors in multivariate analysis regarding DFS (*n* = 78) and OS (*n* = 82). **a**: OS stratified by the NLR; **b**: DFS stratified by the NLR; **c**: DFS stratified by pT status; **d**: DFS stratified by pN status; **e**: OS stratified by age; **f**: DFS stratified by age; **g**: OS stratified by tumour thrombus

The relationships of the independent predictors in multivariate analyses for DFS and OS, such as the preoperative NLR, pT status, pN status, age and tumour thrombus with survival (OS: *n* = 82; DFS: *n* = 78) were investigated, and the results are shown in Fig. 2. Patients with a preoperative higher NLR had a significantly worse rate of survival than those with a lower NLR ratio with regarding both OS and DFS (Mean OS 49.0 months vs 99.7 months, respectively, log-rank *P* = 0.009; Mean DFS 24.5 months vs 90.2 months, respectively, log-rank *P* < 0.001). Patients age > 45 years had a significantly worse rate of survival than those with age ≤ 45 years with regarding both OS and DFS (mean DFS 12.1 months vs 75.0 months, respectively, log-rank *P* = 0.005; mean OS 30.5 months vs 67.8 months, respectively, log-rank *P* = 0.035). Patients at T3-T4 stage and N1 stage had a significantly worse rate of survival than those with at stage T1-T2 and N0 stage regarding DFS (mean DFS 24.5 months vs 90.2 months, respectively, log-rank *P* < 0.001; mean DFS 20.4 months vs 81.9 months, respectively, log-rank *P* < 0.001). Patients positive for a tumour thrombus had a significantly worse rate of survival than those who were negative for a tumour thrombus regarding OS (mean DFS 24.5 months vs 90.2 months, respectively, log-rank *P* < 0.001).

## Discussion

In this multicentre retrospective study, we investigated the prognostic values of the NLR, CRP/Alb ratio, and PLR in 82 Xp11.2 tRCC patients who underwent radical or partial nephrectomy. The results demonstrated that the NLR, CRP/Alb ratio and PLR were all significant predictors and that the NLR was an independent prognostic marker for patients with Xp11.2 tRCC.

Increasing evidence has revealed the involvement of systemic inflammation in cancer development and progression. Neutrophils were shown to produce pro-angiogenic factors such as vascular endothelial growth factor to stimulate tumour development and progression [19]. Moreover, the cytokines involved in cancer-related inflammation, IL-6 and TNFα, may induce neutrophilia [20, 21]. Additionally, relative lymphocytopenia may reflect a lower count of CD4+ T-helper lymphocytes, resulting in a suboptimal lymphocyte-mediated immune response to malignancy [22]. Therefore, an NLR may reflect the combined prognostic information of these two inflammatory factors, and a high NLR has been validated as a poor prognostic factor for several different human cancers [10], including clear cell RCC and non-clear cell RCC [14, 17, 22–24].

C-reactive protein (CRP) is a prototype acute phase protein that was demonstrated to be produced in hepatocytes and regulated by growth factors in the malignant tumors such as IL-6 [25]. An elevated CRP level was reported to be associated with a poorer prognosis in various types of human cancers [25–27]. A study conducted by Guo et al. [16] summarized the potential mechanisms regarding how CRP is associated with cancer in follow several aspects:(1). An increased CRP level is created by the tissue inflammation, which is caused by the tumour growth; (2). Tumour antigens activate the immune responses, leading to increased CRP level; (3). Tumour cells increase CRP expression by producing inflammatory proteins, including CRP or by enhancing the role of inflammatory cytokines such as IL-6 and IL-8, which could indirectly increase the CRP level. More recently, several publications demonstrated that the CRP/Alb ratio could be used to predict the prognosis of several cancers [11, 28, 29], and two additional studies confirmed the prognostic value of the CRP/Alb ratio in RCC patients [16, 30].

Since the possible association between an increased platelet level and cancer metastasis was first described in 1968 [31], an increased PLT level was confirmed to be a prognostic marker for several cancers, including RCC [32, 33]. Furthermore, Emerging evidence has shown that the platelet-to-lymphocyte ratio (PLR) can be used to assess the response to systemic inflammation and RCC prognosis [12, 15, 16].

In this study, we explored the relationships of the NLR, CRP/Alb ratio and PLR with survival in Xp11.2 tRCC patients. Compared with the other systemic inflammatory markers, the NLR, CRP/Alb ratio and PLR had better predictive value for DFS (Fig. 1, Tables 2 and 3). Among of them, the NLR had the highest AUC value (*P* = 0.001). The optimal cut-off value of the NLR was 2.45, which is little lower than the cut-off values of two other studies, whose cut-off values were 2.7 and 3.3, respectively [14, 24]. We consider these differences to be due to the small size of our patients and uniqueness of this tumour. Regarding the CRP/Alb ratio and PLR, the optimal cut-off values were 0.083 and 140, respectively, similar to those reported in previous studies on RCC [15, 16]. Univariate analyses for both DFS and OS showed that a higher NLR, CRP/Alb ratio and PLR were all associated with a poorer prognosis of Xp11.2 tRCC patients (Tables 4 and 5), and multivariate analyses showed that only the NLR independently predicted the DFS of patients with Xp11.2 tRCC (HR: 4.25; 95% CI: 1.19–15.18; *P* = 0.026) along with the pT status (*P* < 0.001), pN status (*P* = 0.019) and age (0.014) (Table 4), and the NLR (HR: 26.26; 95% CI: 1.44–480.3; *P* = 0.028) as well as independently predicted the OS of patients with Xp11.2 tRCC, along with age (*P* = 0.016) and tumour thrombus (*P* = 0.007) (Table 5). Our previous studies on Xp11.2 tRCC confirmed that advanced TNM stage and tumour thrombus are the most significant factors that predict a poor prognosis in Xp11 tRCC [4, 34], and we believe that pT status and N status contribute to the advanced TNM stage in the present study. In addition, Kaplan–Meier survival analysis suggested that the patients with a preoperative higher NLR, T3-T4 stage, N1 stage and age > 45 years had a significantly shorter DFS than those with a lower NLR, T1-T2 stage, N0 stage and age ≤ 45 years, individually. The patients with a higher NLR, age > 45 years and positive for tumour thrombus had a significantly shorter OS than those with a lower NLR, age ≤ 45 years and negative for tumour thrombus. Therefore, a low NLR is associated with the early-stage Xp11.2 tRCC and a high NLR indicates advanced-stage Xp11.2 tRCC, suggesting that the NLR could be a new prognostic indicator related to the progression of Xp11.2 tRCC.

Our findings demonstrate that the NLR, CRP/Alb ratio and PLR were all associated with a poor prognosis in Xp11.2 tRCC patients. Among them, only the NLR independently predicted surgical outcomes of Xp11.2 tRCC patients. These results are important for clinicians to make clinical decisions. According to these preoperative inflammatory markers, patients at high risk can be selected for further treatment and management. With these prognostic factors, more suitable preoperative therapies and more frequent follow-up strategies can be considered for certain high-risk patients with Xp11.2 tRCC.

In addition, the prognostic value of inflammatory markers has never been reported with Xp11.2 tRCC patients. We may, for the first time, predict the surgical outcomes of Xp11.2 tRCC patients using the NLR, CRP/Alb ratio and PLR. Moreover, the sample size of this study was the largest among studies of this tumour worldwide.

To the best of our knowledge, this is the first study that focused on the prognostic values of the NLR, CRP/Alb ratio and PLR in patients with Xp11.2 tRCC. However, this study possesses several limitations. First, our study is a retrospective study, which may limit the prognostic values of the NLR, CRP/Alb ratio and PLR. Therefore, a large-scale prospective study is needed to validate our results. Second, due to the low incidence of Xp11.2 tRCC, our sample size was relatively small, warranting a large-scale study. Third, several other factors that are influential to inflammation such as smoking habits and life styles were not included in the study.

## Conclusions

In summary, we found that the NLR, CRP/Alb ratio and PLR were all potential markers for the survival of Xp11.2 tRCC; thus, they could be considered for clinical decision-making. Among them, the NLR is an independent predictor of both DFS and OS for patients with Xp11.2 tRCC and can be used to predict the surgical outcomes of Xp11.2 tRCC patients who underwent full resection.

## Abbreviations

Alb: Albumin
CRP: C-reactive protein
CRP/Alb: C-reactive protein/albumin
DFS: Disease-free survival
HR: Hazard ratio
LC: Lymphocyte count
LDH: Lactate dehydrogenase level
NC: Neutrophil count
NLR: Neutrophil count to lymphocyte count ratio
OS: Overall survival
PLR: Platelet count to lymphocyte count ratio
PLT: Platelet count
RCC: Renal cell carcinoma
ROC: Receive operating characteristic
TFE3: Transcription factor E3
Xp11.2 tRCC: Xp11.2 translocation/TFE3 gene fusions Renal Cell Carcinoma
